# Clinicopathological characteristics and prognostic factors of invasive micropapillary carcinoma of the breast

**DOI:** 10.1007/s12672-025-04287-0

**Published:** 2025-12-14

**Authors:** Yuxin Qiu, Xiangdi Meng, Xiaolong Chang, Yan Zhang, Furong Hao

**Affiliations:** 1 School of Clinical Medicine, Shandong Second Medical University, Weifang, China; 2 Department of Radiation Oncology, Weifang People’s Hospital, Weifang, China; 3 Department of Radiation Oncology, Gunma University Graduate School of Medicine, Maebashi, Japan; 4 Qilu Hospital of Shandong University (Dezhou Hospital), Dezhou, China

**Keywords:** Breast cancer, Invasive micropapillary carcinoma, Neoadjuvant chemotherapy, Prognostic factors, Clinical outcomes

## Abstract

**Purpose:**

To evaluate the clinicopathological characteristics of breast invasive micropapillary carcinoma (IMPC) and identify factors associated with recurrence and metastasis.

**Methods:**

The study retrospectively analyzed 401 cases of breast IMPC diagnosed between 2017 and 2021. The primary endpoint was recurrence and metastasis–free survival (RMFS). Secondary endpoints included locoregional recurrence–free survival (LRRFS) and distant metastasis–free survival (DMFS). Survival was estimated using the Kaplan–Meier method and compared with the log-rank test. Univariable and multivariable Cox regression analyses were performed to identify prognostic factors. Continuous variables were categorized using maximum log-rank statistics to optimize group stratification.

**Results:**

The median follow-up duration was 46.9 months (range, 1–92 months). The 5-year RMFS, LRRFS and DMFS rates were 86.0% (95% CI, 82.2%-90.0%), 97.5% (95% CI, 95.8%-99.3%) and 86.5% (95% CI, 82.8%-90.5%), respectively. Multivariable Cox analysis showed that maximum tumor diameter (≤ 1.7 cm vs. >1.7 cm, *P* = 0.018), the log odds of positive lymph nodes (LODDS) (≤ 0 vs. >0, *P* = 0.008), histological grade (I-II vs. III, *P* = 0.002), and receipt of neoadjuvant chemotherapy (*P* = 0.003) were independent predictors of recurrence and metastasis.

**Conclusion:**

Breast IMPC carries a relatively high risk of recurrence and metastasis. Tumor size, LODDS, histologic grade, and neoadjuvant chemotherapy were independently associated with outcomes, underscoring the need for closer clinical monitoring and refined risk stratification in this subtype.

**Supplementary Information:**

The online version contains supplementary material available at 10.1007/s12672-025-04287-0.

## Introduction

Invasive micropapillary carcinoma (IMPC) is a relatively rare malignant tumor, accounting for 1% to 8.4% of all breast cancers [1, 2]. Its histologic morphology is distinctive, characterized by small, hollow, or mulberry-like clusters of cancer cells. These tumor cells typically exhibit a characteristic “inside-out” pattern, also known as polarity inversion, in which the apical surface faces the stroma rather than the luminal side [3]. IMPC was first described by Fisher et al. in 1980 as having an “exfoliative appearance” [4], and thirteen years later, Siriaunkgul and Tavassoli formally introduced the term breast invasive micropapillary carcinoma [5]. In 2003, the World Health Organization recognized IMPC as a special subtype of breast cancer. Similar micropapillary features have also been identified in bladder [6], colorectal [7], and lung cancers [8], leading to the broader use of the term invasive micropapillary carcinoma across tumor types [2, 9].

Compared with invasive ductal carcinoma (IDC), breast IMPC is characterized by a markedly higher incidence of lymphovascular invasion (LVI) and lymph node metastasis (LNM) [10, 11]. Due to its core pathological feature of tumor cell polarity inversion, which forms micropapillary or morula-like structures lacking a fibrovascular core, surface glycoproteins are abnormally expressed on the basolateral surface of cells. This leads to reduced intercellular adhesion, facilitating the detachment of tumor cells from the primary lesion and further predisposing it to lymph node metastasis. Recent studies have shown that the log odds of positive lymph nodes (LODDS) serve as an independent prognostic indicator for breast IMPC and improves the accuracy of nodal staging regardless of the absolute number of lymph nodes examined [12]. Although some researchers have suggested that overall survival (OS) in IMPC is comparable to that in IDC despite its aggressive features [13], other study has reported significantly lower 5-year breast cancer-specific survival and recurrence-free survival (RFS) in patients with IMPC [14]. To date, the underlying pathogenesis of IMPC remains unclear, and it is still uncertain whether its distinct clinicopathologic characteristics consistently translate into poorer clinical outcomes.

By analyzing prognostic factors in breast IMPC, this study aims to provide further insight into individualized clinical management and to support future basic research on this unique tumor subtype.

## Materials and methods

### Patients

**Fig. 1:**
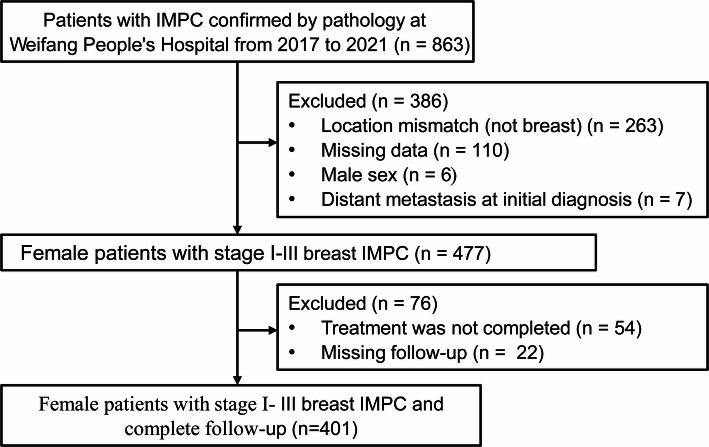
Flow chart of patient selection

This study included general clinical data, clinicopathological and immunohistochemical features, and prognostic information for patients diagnosed with breast IMPC at Weifang People’s Hospital between 2017 and 2021. The inclusion criteria were the following: (1) female patients; (2) patients with IMPC breast cancer confirmed by preoperative biopsy or postoperative pathology; (3) availability of complete general clinical data, pathological data, and follow-up data; and (4) received standard treatment according to clinical guidelines. The exclusion criteria were as follows: (1) male patients; (2) missing information such as degree of dedifferentiation, tumor staging, molecular typing, and treatment methods; (3) distant metastases confirmed at the initial visit; (4) patients with concomitant diagnosis with other cancers; and (5) incomplete follow-up information. The patient selection workflow is presented in Fig. 1. A total of 401 breast IMPC patients were ultimately enrolled (During this period, a total of 5996 patients were diagnosed with breast cancer).

### General clinical data

The following variables were extracted from the medical records: patient age, tumor-node-metastasis (TNM) staging (American Joint Committee on Cancer [AJCC] 8th edition), maximum tumor diameter, lymph node metastasis status (present or absent), and the location and number of metastatic lymph nodes (positive lymph nodes, pLN). The pLN ratio (pLNR) and the LODDS were subsequently calculated. LODDS was defined as the logarithm of the ratio of positive to negative lymph nodes, with 0.01 added to both the numerator and denominator to avoid zero values [12]. The formula was as follows:

$$\:{LODDS}={log}\left(\frac{No.\:PLNs+0.01}{No.\:NLNs+0.01}\right).$$

### Diagnostic criteria

#### Pathological diagnostic criteria

The pathological classification of breast cancer followed the International Classification of Diseases for Oncology, Third Edition. Although the proportion of IMPC components may vary, previous studies have shown that the presence of any identifiable micropapillary component is associated with more aggressive biological behavior [2, 15–17]. Therefore, in this study, all tumors exhibiting micropapillary features were classified as IMPC, regardless of the percentage of micropapillary architecture.

#### Immunohistochemical (IHC) assessment criteria

According to the 2020 American Society of Clinical Oncology/College of American Pathologists (ASCO/CAP) Clinical Practice Guideline Focused Update, tumors with fewer than 1% immunoreactive nuclei were classified as estrogen receptor (ER) or progesterone receptor (PR)-negative, whereas all other cases were considered ER/PR-positive [18]. Given the high rate of ER and PR positivity reported in breast IMPC [12, 19, 20] and to allow for more refined analysis, hormone receptor status was further stratified by average staining intensity. At our institution, staining intensity is visually assessed by dedicated breast pathologists and categorized as negative (−), low positive (+), positive (++), or strong positive (+++), in accordance with the CAP-recommended reporting template for biomarker assessment in breast cancer specimens [21–23].

Human epidermal growth factor receptor 2 (HER2) status was assessed in accordance with the 2018 ASCO/CAP guidelines. HER2 IHC scores of 0 or 1 + were interpreted as negative, whereas a score of 3 + was considered positive. Cases with an IHC score of 2 + were classified as equivocal and underwent further evaluation using dual-probe fluorescence in situ hybridization (FISH) to determine HER2 amplification [24]. For analytical purposes, patients were grouped as HER2-negative (IHC 0 or 1+, or IHC 2 + without amplification) or HER2-positive (IHC 3 + or IHC 2 + with amplification). Patients with hormone receptor–positive or HER2-positive disease received endocrine therapy and anti- HER2 targeted therapy according to standard treatment guidelines.

Ki-67 testing was performed for all invasive breast carcinomas, with positive tumor cells exhibiting brown-yellow nuclear staining. A Ki-67 index of ≤ 14% was defined as low expression.

Molecular subtyping of breast cancer was performed by classifying tumors into four categories: Luminal A-like (ER-positive, PR-positive with high expression, HER2-negative, and low Ki-67 expression); Luminal B, which included a HER2-negative subtype (ER-positive, low PR expression, HER2-negative, and high Ki-67 expression) and a HER2-positive subtype (ER-positive, PR at any level, HER2-positive, and Ki-67 at any level); HER2-overexpressing (ER-negative, PR-negative, HER2-positive, and Ki-67 at any level); and triple-negative breast cancer (TNBC) (ER-negative, PR-negative, HER2-negative, and Ki-67 at any level). Tumors with a luminal-like phenotype that did not meet the criteria for Luminal A-like were classified as Luminal B HER2-negative.

### Follow-up

The duration of follow-up was defined as the interval from the date of pathological diagnosis of IMPC to the last follow-up or the occurrence of local recurrence and/or distant metastasis. Follow-up was conducted primarily through outpatient visits and telephone interviews. The observation period concluded in September 2024. The primary endpoint was recurrence and metastasis–free survival (RMFS), defined as the time from diagnosis to the first documented recurrence or metastasis. Secondary endpoints included locoregional recurrence–free survival (LRRFS) and distant metastasis–free survival (DMFS).

### Statistical methods

Survival curves were generated using the Kaplan–Meier method, and differences between subgroups were compared with the log-rank test. Prognostic factors were evaluated using univariable and multivariable Cox proportional hazards regression models to identify independent predictors of clinical outcomes. Continuous variables were categorized by determining optimal cut-off points based on maximum log-rank statistics. All statistical analyses were performed using R software (version 4.4.0). A P value < 0.05 was considered statistically significant.

### Ethics approval

All procedures were conducted in accordance with the principles of the Declaration of Helsinki (as revised in 2013). This was a retrospective observational study using anonymized patient data. The Institutional Review Board of Weifang People’s Hospital determined that formal ethical approval was not required and waived the requirement for informed consent.

## Results

### Clinicopathological characteristics

This study included 401 patients diagnosed with breast IMPC, with a mean age of 51.8 ± 10.2 years. The clinical characteristics of the cohort are summarized in Table 1. Although T-stage distribution did not differ significantly between the recurrence/metastasis and non–recurrence/metastasis groups (*P* = 0.074), tumors were larger in patients with recurrence or metastasis (median, 2.4 cm [Interquartile range, IQR: 2.0, 3.0] vs. 2.0 cm [IQR: 1.5, 3.0], *P* = 0.043), and N stage was significantly higher in this group (*P* < 0.001).

The resection and metastatic status of sentinel lymph nodes (SLNs) and non-sentinel lymph nodes (NSLNs) were further evaluated. There were no significant differences in the number of SLNs or NSLNs removed intraoperatively between the two groups (all *P* > 0.05). However, the recurrence/metastasis group had a substantially higher median number of metastatic NSLNs compared with the non-recurrence/metastasis group (4 [IQR: 1.0, 14.2] vs. 0 [IQR: 0.0, 5.0], *P* < 0.001).

For the entire cohort, the median number of eLNs during surgery was 18 [IQR: 5, 25], and the median pLNR was 10% [IQR: 0%, 30%]. Although there was no significant difference in eLNs between the recurrence/metastasis and non-recurrence/metastasis groups (*P* = 0.077), the recurrence/metastasis group had a higher median number of pLNs (4.5 vs. 1.0, *P* < 0.001) and a significantly elevated pLNR (30% vs. 10%, *P* < 0.001).

**Table 1:** Patient clinicopathological characteristics (*n* = 401)

Characteristics	All patients	Recurrence/metastasis	Recurrence/metastasis-free	*P* value
	*n* = 401 (%)	*n* = 48 (%)	*n* = 353 (%)	
Age, Mean (SD)	51.8 (10.2)	52.5 (9.6)	51.8 (10.3)	0.659^a^
*pT (AJCC 8th)*				0.074^b^
T1	175 (43.6)	14 (29.2)	161 (45.6)	
T2	208 (51.9)	30 (62.5)	178 (50.4)	
T3	17 (4.2)	4 (8.3)	13 (3.7)	
T4	1 (0.2)	0 (0.0)	1 (0.3)	
*pN (AJCC 8th)*				< 0.001^c^
N0	156 (38.9)	7 (14.6)	149 (42.2)	
N1	103 (25.7)	14 (29.2)	89 (25.2)	
N2	60 (15.0)	7 (14.6)	53 (15.0)	
N3	82 (20.4)	20 (41.7)	62 (17.6)	
*pTNM (AJCC 8th)*				0.001^b^
I	80 (20.0)	3 (6.2)	77 (21.8)	
IIA	124 (30.9)	9 (18.8)	115 (32.6)	
IIB	52 (13.0)	9 (18.8)	43 (12.2)	
IIIA	49 (12.2)	6 (12.5)	43 (12.2)	
IIIB	14 (3.5)	1 (2.1)	13 (3.7)	
IIIC	82 (20.4)	20 (41.7)	62 (17.6)	
Maximum tumor diameter, median [IQR]	2.0 [1.5, 3.0]	2.4 [2.0, 3.0]	2.0 [1.5, 3.0]	0.043^d^
*Location of lymph node metastasis*				0.001^b^
No metastasis	156 (38.9)	7 (14.6)	149 (42.2)	
Axillary region only	158 (39.4)	26 (54.2)	132 (37.4)	
SLN region only	36 (9.0)	4 (8.3)	32 (9.1)	
SC/ICLN region only	2 (0.5)	0 (0.0)	2 (0.6)	
Metastasis in ≥ 2 regions	49 (12.2)	11 (22.9)	38 (10.8)	
Sentinel eLN, median [IQR]	0.0 [0.0, 3.0]	0.0 [0.0, 3.0]	0.0 [0.0, 3.0]	0.083^d^
Sentinel pLN, median [IQR]	0.0 [0.0, 0.0]	0.0 [0.0, 0.0]	0.0 [0.0, 0.0]	0.658 ^d^
Non-sentinel eLN, median [IQR]	17.0 [0.0, 24.0]	19.0 [12.8, 25.2]	17.0 [0.0, 24.0]	0.068^d^
Non-sentinel pLN, median [IQR]	1.0 [0.0, 7.0]	4.0 [1.0, 14.2]	0.0 [0.0, 5.0]	< 0.001^d^
Total eLN, median [IQR]	18.0 [5.0, 25.0]	19.5 [14.8, 25.2]	18.0 [4.0, 24.0]	0.077^d^
pLN, median [IQR]	1.0 [0.0, 7.0]	4.5 [1.0, 14.2]	1.0 [0.0, 5.0]	< 0.001^d^
pLNR, median [IQR]	10% [0%, 30%]	30% [10%, 70%]	10% [0%, 30%]	< 0.001^d^
LODDS, median [IQR]	−1.0 [−3.5, −0.3]	−0.3 [−1.1, 0.3]	−1.1 [−3.5, −0.4]	< 0.001^d^

SD, standard deviation; AJCC, American Joint Committee on Cancer; IQR, interquartile range; SLN, sentinel lymph node; SC/ICLN, supraclavicular and infraclavicular lymph nodes; eLN, number of excised lymph nodes; pLN, number of positive lymph nodes; LODDS, log odds of positive lymph nodes, calculated as: $\:\mathrm{LODDS}=\mathrm{log}(\frac{\mathrm{N}\mathrm{o}.\:\mathrm{P}\mathrm{L}\mathrm{N}\mathrm{s}+0.01}{\mathrm{N}\mathrm{o}.\:\mathrm{N}\mathrm{L}\mathrm{N}\mathrm{s}+0.01}$, where LODDS=0 indicates an equal number of positive and negative lymph nodes excised

^a^ t-test

^b^Fisher’s exact test

^c^Chi-square test

^d^Mann–Whitney U test

As shown in Table 2, the cohort demonstrated a strong tendency toward ER and PR positivity, with a median Ki-67 index of 30% [IQR: 20%, 45%]. In addition, a high proportion exhibited LVI (*n* = 329, 82%) and higher histological grades (grade II: *n* = 236, 58.9%; grade III: *n* = 154, 38.4%). PR rate was significantly higher in the non-recurrence/metastasis group compared with the recurrence/metastasis group (median: 40% [IQR: 2%, 80%] vs. 10% [IQR: 1%, 51.2%]; *P* = 0.004). Histological grade also differed significantly between the two groups (*P* = 0.010). Regarding treatment patterns, most patients underwent mastectomy (*n* = 363, 90.5%) and received adjuvant chemotherapy (*n* = 335, 84.6%). The utilization rate of neoadjuvant chemotherapy (NAC) in breast IMPC was relatively low at 13% (*n* = 52). However, NAC use differed significantly between the recurrence/metastasis and non-recurrence/metastasis groups (*P* = 0.001).

**Table 2:** Pathological, immunohistochemical, and therapeutic characteristics of tumors in patients

Characteristics	All patients	Recurrence/metastasis	Recurrence/metastasis-free	*P* value
	*n* = 401 (%)	*n* = 48 (%)	*n* = 353 (%)	
*ER intensity*				0.199^a^
Negative (−)	53 (13.2)	10 (20.8)	43 (12.2)	
Weakly positive (+)	24 (6.0)	4 (8.3)	20 (5.7)	
Positive (++)	93 (23.2)	7 (14.6)	86 (24.4)	
Strongly positive (+++)	231 (57.6)	27 (56.2)	204 (57.8)	
ER rate, median [IQR]	90.0 [60.0, 95.0]	82.5 [35.0, 90.0]	90.0 [60.0, 95.0]	0.174^b^
*PR intensity*				0.066^a^
Negative (−)	84 (20.9)	12 (25.0)	72 (20.4)	
Weakly positive (+)	30 (7.5)	6 (12.5)	24 (6.8)	
Positive (++)	101 (25.2)	16 (33.3)	85 (24.1)	
Strongly positive (+++)	186 (46.4)	14 (29.2)	172 (48.7)	
PR rate, median [IQR]	40.0 [2.0, 80.0]	10.0 [1.0, 51.2]	40.0 [3.0, 80.0]	0.004^b^
*HER2 status*				0.524^b^
Positive	271 (67.6)	30 (62.5)	241 (68.3)	
Negative	130 (32.4)	18 (37.5)	112 (31.7)	
Ki-67, median [IQR]	30.0 [20.0, 45.0]	25.0 [15.0, 40.0]	30.0 [20.0, 45.0]	0.072^b^
*Molecular subtype*				0.339^c^
HER2 overexpression	48 (12.0)	6 (12.5)	42 (11.9)	
Luminal A-like	30 (7.5)	3 (6.2)	27 (7.6)	
Luminal B (HER2+)	80 (20.0)	11 (22.9)	69 (19.5)	
Luminal B (HER2−)	229 (57.1)	24 (50.0)	205 (58.1)	
TNBC	14 (3.5)	4 (8.3)	10 (2.8)	
*LVI*				0.396^a^
Absent	72 (18.0)	6 (12.5)	66 (18.7)	
Present	329 (82.0)	42 (87.5)	287 (81.3)	
*Histological grade*				0.010^c^
I	11 (2.7)	0 (0.0)	11 (3.1)	
II	236 (58.9)	20 (41.7)	216 (61.2)	
III	154 (38.4)	28 (58.3)	126 (35.7)	
*Neoadjuvant chemotherapy*				
No	349 (87.0)	34 (70.8)	315 (89.2)	0.001^a^
Yes	52 (13.0)	14 (29.2)	38 (10.8)	
*Tumor location*				
Right	177 (44.2)	20 (41.7)	157 (44.6)	0.904^c^
Left	214 (53.5)	27 (56.2)	187 (53.1)	
Bilateral	9 (2.2)	1 (2.1)	8 (2.3)	
Surgical types				0.980^c^
BCS	38 (9.5)	4 (8.3)	34 (9.6)	
MAST	363 (90.5)	44 (91.7)	319 (90.4)	
*Adjuvant chemotherapy*				
No	61 (15.4)	12 (25.5)	49 (14.0)	0.067^a^
Yes	335 (84.6)	35 (74.5)	300 (86.0)	
*Radiotherapy*				0.337^a^
No.	214 (53.4)	22 (45.8)	192 (54.4)	
Yes	187 (46.6)	26 (54.2)	161 (45.6)	

ER, estrogen receptor; PR, progesterone receptor; IQR, interquartile range; HER2, human epidermal growth factor receptor 2; TNBC, triple-negative breast cancer; LVI, lymphovascular invasion; BCS, breast conservation surgery; MAST, Mastectomy

^a^t-test

^b^Fisher’s exact test

^c^Chi-square test

^d^Mann–Whitney U test

### Characteristics of recurrence and metastasis and overall survival in breast IMPC

The median follow-up duration was 46.9 months (range, 1–92 months). During this period, 48 patients (12.0%) developed recurrence or distant metastasis. Local recurrence occurred in 6 cases (5 in the ipsilateral chest wall and 1 in the supraclavicular region). Distant metastasis was documented in 45 patients, with some presenting multiple metastatic sites. The most common sites of metastasis were the bone (*n* = 17), liver (*n* = 13), contralateral breast/axilla/supraclavicular region (*n* = 12), lung (*n* = 7), brain (*n* = 6), mediastinum (*n* = 4), bladder (*n* = 1), and adrenal gland (*n* = 1).

**Fig. 2:**
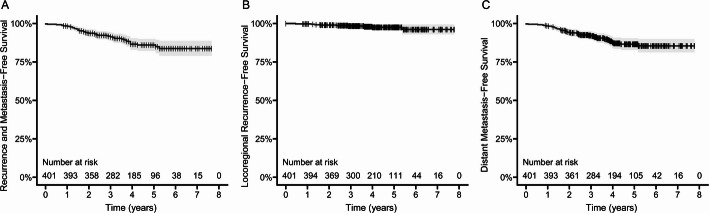
**A** Recurrence and metastasis-free survival, **B** Locoregional recurrence-free survival, and (**C**) Distant metastasis-free survival in breast IMPC patients

The 3-year and 5-year RMFS rates were 91.4% (95% CI, 88.6%−94.2%) and 86.0% (95% CI, 82.2%−90.0%), respectively (Fig. 2A). The 3-year and 5-year LRRFS rates were 98.4% (95% CI, 97.1%−99.7%) and 97.5% (95% CI, 95.8%−99.3%), respectively (Fig. 2B). The 3-year and 5-year DMFS rates were 92.2% (95% CI, 89.6%–95.0%) and 86.5% (95% CI, 82.8%−90.5%), respectively (Fig. 2C).

### Cox regression analysis of factors associated with recurrence and metastasis in breast IMPC

Cox univariable and multivariable regression analyses were performed to identify factors associated with recurrence and metastasis in breast IMPC (Table 3). Continuous variables—including age, tumor size, number of pLN, and ER/PR positivity—were dichotomized using cut-off values determined by maximum log-rank statistics to facilitate clinical interpretation and comparison. In addition, to avoid redundancy and multicollinearity, only one variable was included in the analyses when multiple variables conveyed similar clinical meaning.

In univariable Cox regression, maximum tumor diameter > 1.7 cm, > 4 pLNs, LODDS > 0 (indicating a higher ratio of positive to negative lymph nodes), histological grade III, and receipt of NAC were all significant risk factors for recurrence and metastasis (all *P* < 0.05). In contrast, PR positivity > 50% was a protective factor (*P* = 0.009). Multivariable Cox regression identified maximum tumor diameter (≤ 1.7 cm vs. >1.7 cm; HR = 2.68, 95% CI, 1.18–6.06; *P* = 0.018), LODDS (≤ 0 vs. >0; HR = 3.16, 95% CI, 1.36–7.34; *P* = 0.008), histological grade (I-II vs. III; HR = 2.64, 95% CI, 1.44–4.87; *P* = 0.002), and NAC (No vs. Yes; HR = 2.77, 95% CI, 1.41–5.43; *P* = 0.003) as independent predictors of recurrence and metastasis in breast IMPC.

**Table 3:** Cox regression analysis of factors associated with recurrence and metastasis in patients with breast IMPC

Characteristics	Univariable analysis		Multivariable analysis	
	HR (95% CI)	*P* value	HR (95% CI)	*P* value
Age (≤ 40 vs. >40)	1.71 (0.61–4.77)	0.304		
Maximum tumor diameter (≤ 1.7 cm vs. >1.7 cm)	2.86 (1.28–6.38)	0.010	2.68 (1.18–6.06)	0.018
Number of pLNs (≤ 4 vs. >4)	2.38 (1.35–4.20)	0.003	0.88 (0.38–2.04)	0.764
LODDS (≤ 0 vs. >0)	3.79 (2.15–6.68)	< 0.001	3.16 (1.36–7.34)	0.008
ER positivity rate (≤ 50% vs. >50%)	0.76 (0.40–1.45)	0.409		
PR positivity rate (≤ 50% vs. >50%)	0.43 (0.23–0.81)	0.009	0.67 (0.35–1.30)	0.240
HER2 (Negative vs. Positive)	1.25 (0.70–2.25)	0.449		
Ki-67 (≤ 14% vs. >14%)	0.85 (0.38–1.91)	0.700		
LVI (Absent vs. Present)	1.49 (0.63–3.52)	0.358		
Histological grade (I-II vs. III)	2.58 (1.45–4.59)	0.001	2.64 (1.44–4.87)	0.002
NAC (No vs. Yes)	2.98 (1.60–5.56)	0.001	2.77 (1.41–5.43)	0.003
Surgical types (BCS vs. MAST)	0.97 (0.35–2.71)	0.956		
Adjuvant chemotherapy (No vs. Yes)	0.53 (0.27–1.02)	0.058		
Radiotherapy (No vs. Yes)	1.39 (0.79–2.45)	0.259		

LODDS (log odds of positive lymph nodes) is calculated as $\:\mathrm{LODDS}=\mathrm{log}(\frac{No.\:PLNs+0.01}{No.\:NLNs+0.01})$, where LODDS = 0 indicates an equal number of positive and negative lymph nodes excised.

ER, estrogen receptor; PR, progesterone receptor; HR, hazard ratio; CI, confidence interval, pLNs, positive lymph nodes; HER2, human epidermal growth factor receptor 2; LVI, lymphovascular invasion; NAC, neoadjuvant chemotherapy; BCS, breast conservation surgery; MAST, Mastectomy

### Survival analysis of independent prognostic factors for recurrence and metastasis in breast IMPC

**Fig. 3:**
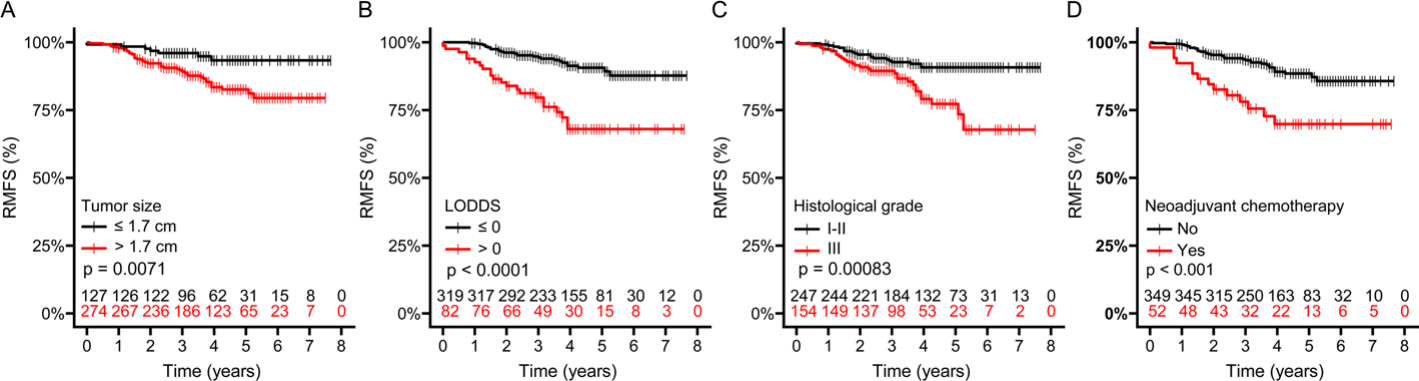
Comparison of recurrence and metastasis-free survival (RMFS) in breast IMPC patients based on tumor size, LODDS, histological grade, and neoadjuvant chemotherapy. LODDS: Log odds of positive lymph nodes, calculated as: $\:\mathrm{LODDS}=\mathrm{log}\left(\frac{\mathrm{N}\mathrm{o}.\:\mathrm{P}\mathrm{L}\mathrm{N}\mathrm{s}+0.01}{\mathrm{N}\mathrm{o}.\:\mathrm{N}\mathrm{L}\mathrm{N}\mathrm{s}+0.01}\right)$, where LODDS=0 indicates an equal number of excised positive lymph nodes (PLNs) and negative lymph nodes (NLNs)

The risk of recurrence and metastasis in breast IMPC was further evaluated in subgroups defined by tumor size, LODDS, histological grade, and NAC status. Patients with a maximum tumor diameter > 1.7 cm had a significantly lower 5-year RMFS than those with tumors ≤ 1.7 cm (82.6% [95% CI, 77.6%−87.9%] vs. 93.3% [95% CI, 88.5%−98.4%], *P* = 0.007) (Fig. 3A). In the LODDS subgroup analysis, patients with LODDS ≤ 0 had markedly better RMFS compared with those with LODDS > 0 (5-year RMFS: 90.6% [95% CI, 87.0%−94.4%] vs. 68.0% [95% CI, 57.4%−80.6%], *P* < 0.001), indicating that a higher proportion of negative lymph nodes was associated with a lower risk of recurrence and metastasis (Fig. 3B). Patients with histological grade III tumors also demonstrated significantly poorer outcomes compared with those with grade I-II tumors (5-year RMFS: 77.3% [95% CI, 69.4%−86.1%] vs. 90.7% [95% CI, 86.9%−94.8%], *P* < 0.001) (Fig. 3C). Notably, NAC did not reduce the risk of recurrence or metastasis in breast IMPC. Instead, patients who received NAC had significantly worse prognoses (5-year RMFS: 69.8% [95% CI, 57.5%−84.8%] vs. 88.5% [95% CI, 84.6%−92.5%] in the non-NAC group, *P* < 0.001) (Fig. 3D).

### Correlation analysis and comparison of lymph node metastasis-related indicators

**Fig. 4:**
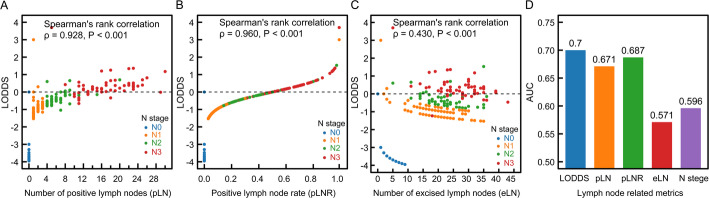
Spearman correlation analysis between LODDS and lymph node-related indicators and their predictive performance for recurrence and metastasis in breast IMPC. Note: LODDS, log odds of positive lymph nodes, calculated as: $\:\mathrm{LODDS}=\mathrm{log}\left(\frac{\mathrm{N}\mathrm{o}.\:\mathrm{P}\mathrm{L}\mathrm{N}\mathrm{s}+0.01}{\mathrm{N}\mathrm{o}.\:\mathrm{N}\mathrm{L}\mathrm{N}\mathrm{s}+0.01}\right)$. LODDS = 0 says that the number of excised positive lymph nodes (PLN) equals the number of excised negative lymph nodes (NLN)

The associations between LODDS and other lymph node–related variables (pLN, pLNR, eLN, and N stage) were examined, and their predictive value for recurrence and metastasis in breast IMPC was evaluated. LODDS was strongly correlated with the number of pLNs (Spearman’s rank correlation test, ρ = 0.928, *P* < 0.001), pLNR (ρ = 0.960, *P* < 0.001), and N stage (ρ = 0.919, *P* < 0.001), whereas its correlation with the eLNs was weaker (ρ = 0.430, *P* < 0.001) (Fig. 4A, C). Among the lymph node–related indicators, LODDS demonstrated the highest predictive value for recurrence and metastasis, with an AUC of 0.70 (Fig. 4D).

### Subgroup analysis of the impact of neoadjuvant chemotherapy on breast IMPC

**Fig. 5:**
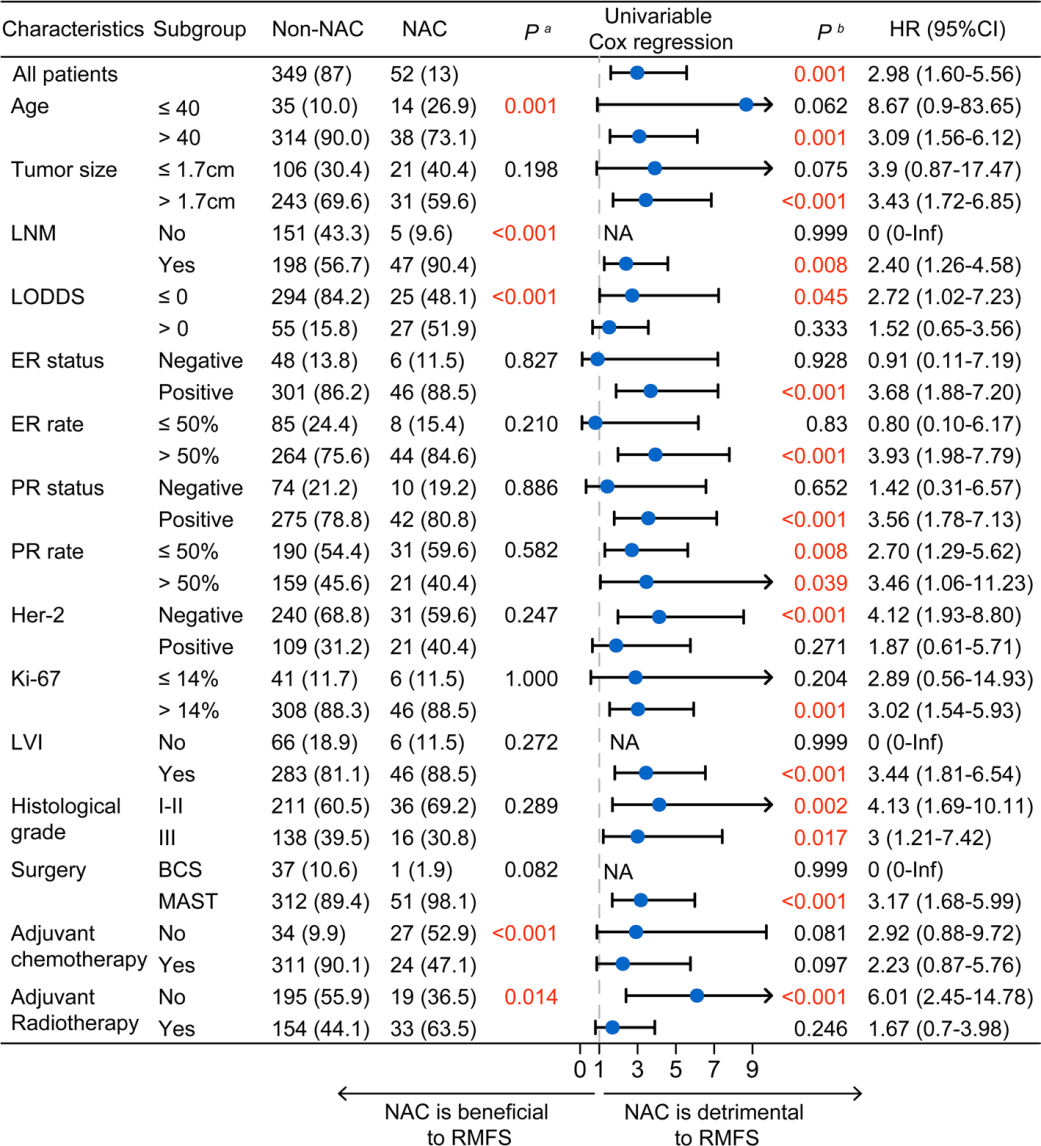
Subgroup analysis of neoadjuvant chemotherapy. ^a^p-value from chi-square test; ^b^p-value from univariable Cox regression

Further subgroup analyses were performed to evaluate the impact of NAC on recurrence and metastasis in patients with breast IMPC. Overall, NAC was associated with worse prognostic outcomes, and no subgroup demonstrated a survival benefit with NAC use. As shown in Fig. 5, patients who received NAC were generally younger and had higher rates of LNM. They were also more likely to receive radiotherapy rather than adjuvant chemotherapy (all *P* < 0.05). Significant differences were observed between the NAC and non-NAC groups in age, LN status, LODDS, adjuvant chemotherapy, and radiotherapy (Chi-squared test, all *P* < 0.05).

Propensity score matching (PSM) was subsequently performed using the nearest-neighbor method with a caliper width of 0.01 to balance these baseline characteristics. After matching, the two cohorts showed no significant differences in baseline variables (all *P* > 0.05; Supplementary Table 1). However, a statistically significant survival difference persisted in favor of the non-NAC group (log-rank test, *P* = 0.038; Supplementary Fig. 1).

### Risk stratification of breast IMPC using four independent prognostic factors

**Fig. 6:**
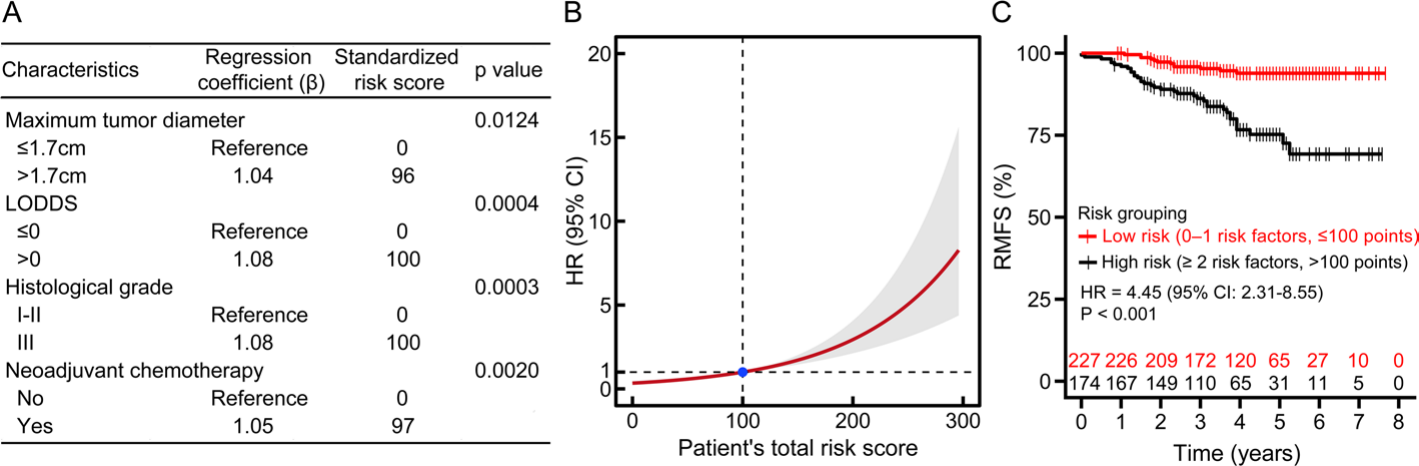
**A** Risk score, **B** Risk stratification, and **C** Stratified assessment based on multivariable Cox regression analysis. LODDS, log odds of positive lymph nodes, calculated as: $\:\mathrm{LODDS}=\mathrm{log}(\frac{\mathrm{N}\mathrm{o}.\:\mathrm{P}\mathrm{L}\mathrm{N}\mathrm{s}+0.01}{\mathrm{N}\mathrm{o}.\:\mathrm{N}\mathrm{L}\mathrm{N}\mathrm{s}+0.01})$, LODDS = 0 indicates that the number of excised positive lymph nodes (PLNs) equals the number of excised negative lymph nodes (NLNs)

A prognostic risk model for breast IMPC was constructed based on the four independent predictors identified in the multivariable Cox regression analysis (Table 3): maximum tumor diameter, LODDS, histological grade, and NAC status. Regression coefficients were converted into individual risk scores, and a total risk score was calculated for each patient (Fig. 6A). Using restricted cubic splines (RCS), the optimal cut-off for the total risk score was determined to be 100 points (corresponding to HR = 1), and this threshold was used for risk stratification (Fig. 6B).

Patients with a total risk score ≤ 100 points were classified into the low-risk group (*n* = 227; defined as having 0–1 risk factor), whereas those with scores > 100 points were classified into the high-risk group (*n* = 174; defined as having ≥ 2 independent risk factors). Kaplan–Meier analysis demonstrated that the high-risk group had a significantly greater likelihood of recurrence and metastasis than the low-risk group (HR = 4.45; 95% CI, 2.31–8.55; *P* < 0.001) (Fig. 6C).

## Discussion

Breast IMPC is characterized by a markedly high incidence of LVI and LNM, features that contribute to its aggressive biological behavior and tendency toward early recurrence and distant spread [10, 16, 25–27]. However, strategies to mitigate the risk of recurrence and metastasis in IMPC remain debated. In this study, tumor size > 1.7 cm, pLNs > 4, LODDS > 0, histological grade III, and receipt of NAC were identified as significant risk factors for recurrence and metastasis. Compared with IDC, breast IMPC is consistently reported to have a higher proportion of LNM [22, 28–30]. Elzohery et al. conducted a comparative analysis of IMPC and IDC and found that the LNM rate was markedly higher in IMPC (68.8% vs. 56%) [22]. A key mechanism underlying this phenomenon is the reversal of tumor cell polarity [31]. This polarity inversion results in mucins—normally secreted into glandular lumina—appearing on the external surfaces of cell clusters, a defining histological feature of IMPC. Alterations in cell polarity or epithelial–mesenchymal transition further facilitate tumor invasiveness and metastatic potential [22]. Among lymph node–related metrics, LODDS demonstrated superior performance in predicting recurrence and metastasis compared with other indicators. Although the total number of excised sentinel and non-sentinel lymph nodes did not differ significantly between the recurrence/metastasis and non-recurrence groups, patients who developed recurrence or metastasis showed a substantially higher burden of metastatic non-sentinel lymph nodes. This observation underscores the importance of adequate lymph node assessment. Clinicians—particularly surgeons—should consider comprehensive lymph node dissection when indicated to improve the detection of positive nodes and thereby potentially reduce the risk of recurrence and metastasis [10, 12, 20].

At present, there is no standardized treatment protocol specifically for IMPC, and therapeutic strategies are generally extrapolated from those used for IDC. Surgical resection remains the primary treatment modality. Given the high propensity of IMPC for local recurrence, several authors have advocated more extensive surgical approaches, including total mastectomy with axillary lymph node dissection and wider surgical margins [19, 20, 32]. However, existing studies indicate that although radical or modified radical mastectomy is frequently performed in IMPC, these more aggressive surgical approaches do not translate into improved prognosis. In our cohort, patients tended to present with a higher burden of lymph node metastasis despite relatively small tumor size and lower T stage at diagnosis. Most IMPC cases were treated with modified radical mastectomy (*n* = 363, 90.5%). Consistent with previous reports [33], our findings demonstrated no significant difference in prognosis between patients undergoing breast-conserving surgery and those receiving modified radical mastectomy. These results suggest that the extent of breast surgery may not be the primary determinant of long-term outcomes in IMPC.

In this study, only 52 patients (13%) received preoperative NAC; however, a significant difference in NAC utilization was observed between the recurrence/metastasis and non-recurrence/metastasis groups. Both univariable and multivariable Cox regression analyses identified NAC as an independent risk factor for recurrence or metastasis. Although NAC can suppress tumor cell proliferation and induce apoptosis, subgroup analyses of the present cohort showed no recurrence-free survival benefit in IMPC. Instead, NAC was associated with a higher risk of recurrence or metastasis, with substantially poorer 5-year RMFS in the NAC group compared with the non-NAC group (69.8% vs. 88.5%, *P* < 0.001). Importantly, this survival difference persisted even after propensity score matching, which balanced key baseline characteristics between the two groups. Recent research supports these findings particularly in TNBC subtypes [34]. Additionally, Doublier et al. reported that MCF-7 three-dimensional spheroids upregulated P-glycoprotein expression through HIF-1 activation [35], resulting in doxorubicin resistance—an observation that may help explain the clinical chemoresistance observed in IMPC.

The median follow-up time in this study was 46.9 months, during which 48 patients (12.0%) developed recurrence or distant metastasis. Among these, 6 cases represented locoregional recurrence (5 involving the ipsilateral chest wall and 1 in the supraclavicular region), while 45 cases were distant metastases. Several patients exhibited multiple metastatic sites, most commonly the bone and liver. Bone metastasis is well recognized as the predominant metastatic pattern in IMPC [36],, potentially linked to the high expression of bone morphogenetic proteins in tumor cells [37, 38] and activation of the RANKL signaling pathway [39, 40]. Metastasis to the contralateral breast, axilla, or supraclavicular region was also frequently observed. Additional metastatic sites included the lung, brain, and mediastinum, with rare involvement of the bladder and adrenal gland—patterns consistent with previous reports [1, 41]. A propensity score–matched analysis conducted by Ye et al. demonstrated that, compared with invasive IDC, IMPC exhibits similar overall survival but a shorter RFS [13].

Several limitations of this study should be acknowledged. First, overall survival was not analyzed, and the influence of radiotherapy dose, field design, and specific chemotherapy regimens on prognosis was not assessed. Second, imbalances in treatment-related variables—including the predominance of mastectomy and variations in chemotherapy and radiotherapy use—may have introduced selection bias and potentially confounded the findings. In addition, the semi-quantitative, intensity-based assessments of ER and PR staining may introduce some degree of interobserver variability, although standardized institutional templates were used. Despite these limitations, the present study contributes meaningful evidence toward understanding and reducing the risk of recurrence and metastasis in patients with breast IMPC, with the aim of ultimately alleviating the clinical burden of this disease.

## Conclusion

In breast IMPC, maximum tumor diameter > 1.7 cm, LODDS > 0, histologic grade III, and neoadjuvant chemotherapy were associated with increased risk of recurrence and metastasis, whereas high PR expression (> 50%) was protective. LODDS demonstrated superior prognostic performance compared with traditional lymph node metrics, supporting its utility in improving risk stratification for breast IMPC. Further multicenter studies are needed to validate these results and refine prognostic models for this rare breast cancer subtype.

## Supplementary Information

Supplementary Material 1.

## Data Availability

The data could be obtained by contacting the corresponding author.
