# Supplementary material for: Clinicopathological characteristics and prognostic factors of invasive micropapillary carcinoma of the breast
Source: Discov Oncol. 2025 Dec 14;17:112. doi: 10.1007/s12672-025-04287-0 (PMC12819910; doi:10.1007/s12672-025-04287-0)
Supplement: Supplementary file 1 — Supplementary Material 1. [file 12672_2025_4287_MOESM1_ESM.docx]

**Supplementary table 1.** Patient characteristics in the NAC group and non-NAC group after PSM

| Characteristics | Non-NAC | NAC | *P value ^a^* |
| --- | --- | --- | --- |
|  | n = 37 (%) | n = 37 (%) |  |
| Age |  |  | 1.000 |
| ≤ 40 | 7 (18.9) | 7 (18.9) |  |
| > 40 | 30 (81.1) | 30 (81.1) |  |
| Tumor size |  |  | 0.634 |
| ≤ 1.7cm | 13 (35.1) | 16 (43.2) |  |
| > 1.7cm | 24 (64.9) | 21 (56.8) |  |
| Lymph node metastasis |  |  | 0.097 |
| No | 12 (32.4) | 5 (13.5) |  |
| Yes | 25 (67.6) | 32 (86.5) |  |
| LODDS |  |  | 1.000 |
| ≤ 0 | 22 (59.5) | 22 (59.5) |  |
| > 0 | 15 (40.5) | 15 (40.5) |  |
| ER status |  |  | 0.734 |
| Negative | 6 (16.2) | 4 (10.8) |  |
| Positive | 31 (83.8) | 33 (89.2) |  |
| ER rate |  |  | 0.563 |
| ≤ 50% | 9 (24.3) | 6 (16.2) |  |
| > 50% | 28 (75.7) | 31 (83.8) |  |
| PR status |  |  | 1.000 |
| Negative | 8 (21.6) | 8 (21.6) |  |
| Positive | 29 (78.4) | 29 (78.4) |  |
| PR rate |  |  | 1.000 |
| ≤ 50% | 23 (62.2) | 23 (62.2) |  |
| > 50% | 14 (37.8) | 14 (37.8) |  |
| HER2 |  |  | 0.478 |
| Negative | 24 (64.9) | 20 (54.1) |  |
| Positive | 13 (35.1) | 17 (45.9) |  |
| Ki-67 |  |  | 1.000 |
| ≤ 14% | 3 (8.1) | 2 (5.4) |  |
| > 14% | 34 (91.9) | 35 (94.6) |  |
| LVI |  |  | 1.000 |
| No | 6 (16.2) | 6 (16.2) |  |
| Yes | 31 (83.8) | 31 (83.8) |  |
| Histological grade |  |  | 0.456 |
| I-II | 23 (62.2) | 27 (73.0) |  |
| III | 14 (37.8) | 10 (27.0) |  |
| Surgery |  |  | 0.354 |
| BCS | 4 (10.8) | 1 (2.7) |  |
| Mastectomy | 33 (89.2) | 36 (97.3) |  |
| Adjuvant chemotherapy |  |  | 1.000 |
| No | 13 (35.1) | 13 (35.1) |  |
| Yes | 24 (64.9) | 24 (64.9) |  |
| Radiotherapy |  |  | 1.000 |
| No | 17 (45.9) | 17 (45.9) |  |
| Yes | 20 (54.1) | 20 (54.1) |  |

^a^: P-value from the chi-square test. Abbreviations: PSM, propensity score matching (method: nearest; caliper value: 0.01). NAC, neoadjuvant chemotherapy; LODDS, LODDS, log odds of positive lymph nodes, calculated as: $LODDS=log (\frac{No. PLNs+0.01}{No. NLNs+0.01}$, where LODDS=0 indicates an equal number of positive and negative lymph nodes excised. ER, estrogen receptor; PR, progesterone receptor; BCS, Breast-conserving surgery; LVI, Lymphovascular invasion


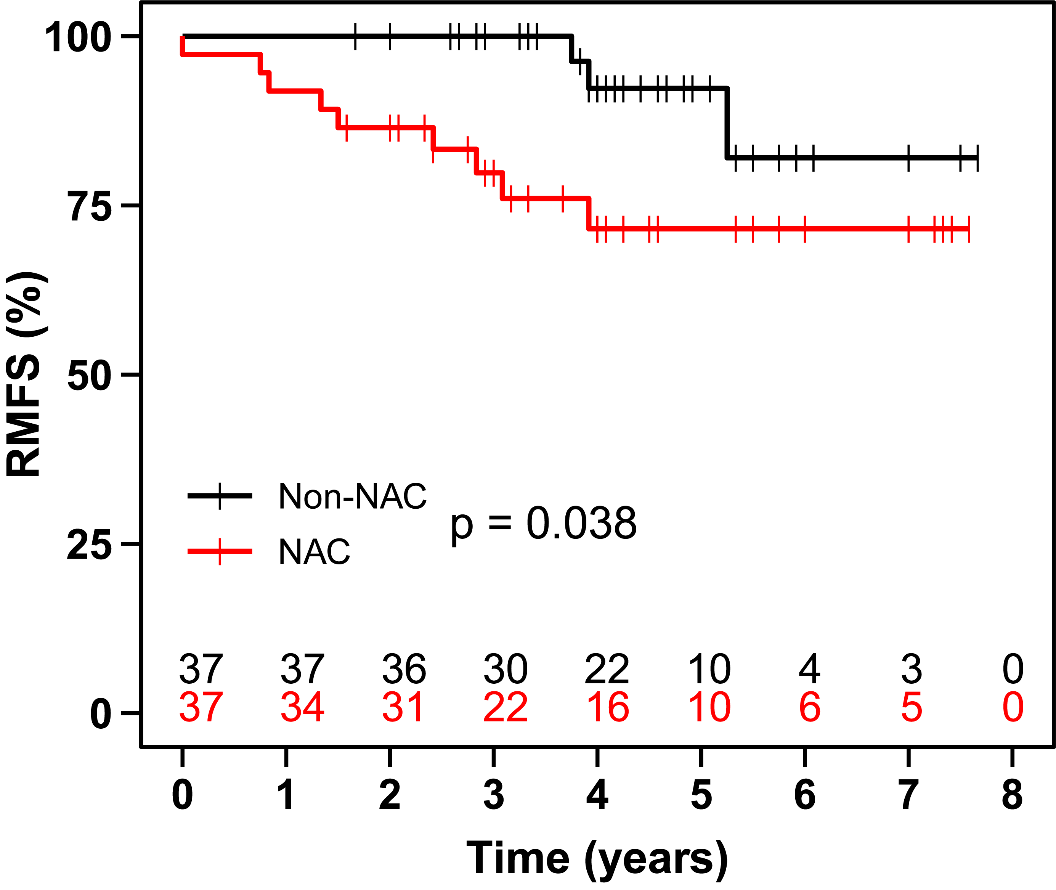


**Supplementary figure 1.** Kaplan-Meier method curves of RMFS in the NAC group and non-NAC group after PSM. Abbreviations: RMFS: recurrence and metastasis-free survival; NAC: neoadjuvant chemotherapy; PSM, Propensity Score Matching.
